# Assessing Public Awareness of the Malaria Vaccine in Sub-Saharan Africa

**DOI:** 10.3390/tropicalmed7090215

**Published:** 2022-08-30

**Authors:** Taiwo Opeyemi Aremu, Chinar Singhal, Oluwafemi Augustine Ajibola, Emmanuel Agyin-Frimpong, Akua Asantewaa Appiah-Num Safo, Maduabuchi Romanus Ihekoronye, Stella Esther Nabirye, Olihe Nnenna Okoro

**Affiliations:** 1 Department of Pharmaceutical Care & Health Systems (PCHS), College of Pharmacy, University of Minnesota, 308 Harvard Street SE, Minneapolis, MN 55455, USA; 2 Division of Environmental Health Sciences, School of Public Health, University of Minnesota, 420 Delaware Street SE, Minneapolis, MN 55455, USA; 3 Division of Epidemiology & Community Health, School of Public Health, University of Minnesota, 300 West Bank Office Building, 1300 S. 2nd Street, Minneapolis, MN 55454, USA; 4 Department of Pulmonary/Critical Care, Louisiana State University Health Sciences Center New Orleans, 1901 Perdido Street, Suite 3205, New Orleans, LA 70112, USA; 5 Department of Medicine, Korle-Bu Teaching Hospital, Guggisberg Avenue, Accra KB77, Ghana; 6 Department of Clinical Pharmacy and Pharmacy Administration, Faculty of Pharmacy, Obafemi Awolowo University, Ile-Ife 220101, Nigeria; 7 Department of Immunology and Molecular Biology, Makerere University College of Health Sciences, Old Mulago Hill Road, Kampala 7072, Uganda; 8 Department of Pharmacy Practice & Pharmaceutical Sciences, College of Pharmacy, University of Minnesota, Duluth, MN 55812, USA

**Keywords:** malaria, infectious disease, awareness, vaccine, sub-Saharan Africa

## Abstract

Background: Malaria infection remains one of the leading causes of death in sub-Saharan Africa. Over the years, several measures have been implemented for the prevention of malaria, including vector control with insecticide-treated nets, indoor residual spraying, and seasonal or traveling prophylactics. In 2021, the World Health Organization (WHO) approved the use of the malaria vaccine in children. We conducted a cross-sectional survey study in three sub-Saharan African countries—Uganda, Ghana, and Nigeria—to assess public awareness of the malaria vaccine among the residents of these countries. Method: A cross-sectional, web-based survey was conducted between time January 2022 and April 2022 using Qualtrics^®^ software (Version number: April 2022; Qualtrics, Provo, UT, USA). A total of 3896 responses were analyzed using SAS OnDemand for Academics software. Linear regression model was used to assess the relationship between the demographic characteristics and awareness of the malaria vaccine, using a level of significance (alpha) of 0.05. Result: Overall, there was significant association between the level of education and public awareness of the malaria vaccine in each of the countries studied. Gender and place of residence were associated with awareness in Nigeria and Uganda, while younger respondents were more likely to be aware of the malaria vaccine in Ghana. Conclusion: Given the negative impact of lack of awareness and knowledge, misinformation and conspiracy theories on immunization programs, public health campaigns preceding the population-wide roll-out of the novel malaria vaccine should target the less-educated, and those residing in more rural areas, while assuring equitable access to the malaria vaccine across sub-Saharan Africa.

## 1. Introduction

Malaria is an infectious disease of enormous public health importance, particularly in sub-Saharan Africa. It is caused by *Plasmodium* parasites transmitted to humans through the bites of infected female *Anopheles* mosquitoes. Reports from the World Health Organization (WHO) indicate that, in 2020, malaria was responsible for 627,000 deaths worldwide, out of which the WHO sub-Saharan Africa region accounted for about 96% [1].

For a more effective control of malaria, RTS, S/AS01, a four-dose malaria vaccine was approved for use by the WHO in October 2021 following a phase III efficacy trial in seven sub-Saharan African countries [2,3]. The four-dose schedule demonstrated 25.9% efficacy (95% CI, 19.9 to 31.5) in a cohort of 6537 children between the ages of 6–12 weeks and 36.3% (95% CI, 31.8 to 40.5) among a study population of 8922 5–17 month-old children, with clinical malaria. The first three doses are administered intramuscularly at 5–9 months, while the fourth dose is recommended at 15–18 months of age [3]. During the pilot study in Ghana, the malaria vaccine was found to prevent approximately 4 in 10 malaria cases and 3 in 10 life-threatening severe malaria cases [4].

While anticipating the country-level roll-out of the distribution and administration of the malaria vaccine in sub-Saharan African countries, we conducted a cross-sectional survey with the aim of assessing the level of public awareness of the malaria vaccine among residents of sub-Saharan African countries, specifically Uganda, Ghana, and Nigeria.

### 1.1. Uganda

In Uganda, malaria is highly endemic in approximately 95% of the country which corresponds to approximately 33 million exposed individuals in the population [5]. According to the World Health Organization 2021 report, Uganda accounted for 3.5% of the deaths by malaria globally [6].

There are three ongoing strategic control interventions that have been implemented since 2014 to curb malaria infection and transmission. These include long-lasting insecticide-treated bed nets; indoor residual spraying and larval source management; and drug therapy for more efficient disease treatment [7]. In 2004, increasing treatment failure observed with chloroquine/sulfadoxine treatment resulted in a change in the treatment policy to artemisinin-based combination therapy as the first-line treatment for uncomplicated malaria, which has played a key role in curbing the disease burden [7]. However, there is emerging evidence of artemisinin-resistant *P falciparum* in Africa [8], necessitating a more effective strategy for reducing the malaria burden.

### 1.2. Ghana

Malaria is endemic in Ghana with a perennial pattern of peak and seasonal variations relative to the geographic regional location [9]. According to the WHO 2021 report, Ghana accounted for 2.1% of all malaria cases and 1.9% of deaths from malaria globally, in 2020 [6].

There have been major attempts over the decades to address malaria morbidity and mortality through national policies. In 1999, the Roll Back Malaria project was mainly launched to make treatment and preventive strategies of malaria nationally available, culminating in the development of the first National Malaria Strategic Plan (2000–2010), with the aim of reducing specific morbidity and mortality by 50% by the year 2010 [10,11]. This signaled the introduction of new and effective interventions such as the treatment of uncomplicated malaria using artemisinin-based combination therapy, malaria prevention in pregnancy using sulfadoxine/pyrimethamine and indoor residual spraying of insecticide.

The strategic plan for 2014–2020 had the overall goal of reducing the malaria morbidity and mortality burden by 75% by the year 2020, which was interestingly realized [9,12]. The main priority program areas included strategies for prevention; diagnosis and treatment; and surveillance, monitoring, and evaluation.

In 2019, the malaria vaccine pilot was officially rolled out in Ghana as one of three countries (along with Kenya and Malawi) where the RTS,S, vaccine was made available to children up to 2 years of age [4]. The phased malaria vaccine is currently administered in selected areas where the proportion of people infected with malaria is at least 20%, childhood vaccination is high, and there are adequate numbers of children at the right age to receive the vaccine. The regions currently enrolled include Brong Ahafo, Central and Volta regions and other upper east regions.

### 1.3. Nigeria

Nigeria has the highest burden of malaria (26.8%) in Africa [1]. Accounting for 31.9% of malaria deaths in 2020, Nigeria is the highest contributor to malaria mortality; with 80% of these deaths occurring among children aged 5 years and below [6].

Malaria continues to be an economic burden to individuals, households, and the entire Nigeria health system [13,14,15]. The emergence of resistance to commonly used antimalarial medications has also added to the economic cost of treatment [16,17].

To slow the emergence of antimalarial resistance, the WHO recommended that malaria treatment only be reserved for individuals with a positive parasitological diagnosis [18]. The Nigeria National Malaria Control Program therefore introduced the “test before treatment” policy [19]. However, due to slow system-wide adoption by 2017, the prevalence of testing in febrile children below 5 years of age was only 14% [20]. Other measures for the prevention and control of malaria include the use of long-lasting insecticide-treated nets, intermittent preventive treatment during pregnancy, vector spraying, and improved environmental hygiene among other similar interventions [21,22].

In anticipation of the malaria vaccine, a recent study among caregivers in Southeastern Nigeria established an awareness level of 48.2% and 88.2% of respondents had a positive perception of the vaccine [23]. The prospects for local capacity in vaccine production was recently boosted by the WHO approval for Nigeria alongside five other African countries including Egypt, Kenya, Senegal, South Africa, and Tunisia, to receive necessary technological support to produce vaccines [24].

The study objective was to assess the level of public awareness of the malaria vaccine in sub-Saharan African countries (Uganda, Ghana, and Nigeria).

## 2. Materials and Methods

The current study was exploratory with a cross-sectional design which used a web-based survey for data collection. This design was deemed appropriate because of its ability to reach a wide spectrum of relevant respondents across the study sites within a short time frame so as to generate a “snapshot” of the situation. The study sites (Nigeria, Ghana, and Uganda) are typical holoendemic settings for malaria, hence data from these locations were considered appropriate representations of the epidemiological portrait of malaria in sub-Saharan Africa. In view of the online data collection strategy, the study settings provided a sufficient population of respondents due to the relatively high penetration of mobile telephony in these countries. A convenience sampling technique was employed as any adult (≥18 years of age) who was consenting and able to respond electronically to the questionnaire was invited to take the survey.

Data were collected using a questionnaire developed by the investigators following detailed review of relevant literature. Items of the instrument sought information on the demographic characteristics of respondents, knowledge of malaria and its treatment and preventative measures, awareness of the malaria vaccine, and its acceptability for administration in children.

The Qualtrics^®^ software (Version number: April 2022; Qualtrics, Provo, UT, USA) was used for data collection. The questionnaire was anonymously self-administered, using a link which was shared with residents of the three sub-Saharan African countries (Uganda, Ghana, and Nigeria), through social media platforms and text messaging apps such as WhatsApp, Facebook, Instagram, Twitter, and Telegram. The data were collected over an 11-week period between January and April 2022. Individuals aged 18 and above and living in any of the three sub-Saharan African countries at the time of survey were eligible to participate in this study. No remuneration was offered to the respondents. However, at the end of the survey, participants had the option to provide their email address or phone number via a different survey link, to enter into a drawing for one of twenty USD 20 cash prizes.

The University of Minnesota Institutional Review Board approved this study (IRB ID: STUDY00014409). All participants provided informed consent before taking the survey.

### Data Analysis

The dataset originally had 4265 observations. Observations with missing relevant data (*n* = 369) were excluded, bringing it down to 3896 observations with data on the covariates of interest for the study. These were included in the analysis.

The outcome of interest was being aware of the malaria vaccine or not. This was categorized into two options: Yes or No. The question asked in the survey was: “Have you heard about the malaria vaccine; an immunization that can help prevent malaria?”

Data were cleaned and analyzed using SAS OnDemand for Academics software. Descriptive data analysis was first performed to summarize the data. Second, the relationship between the demographic characteristics and awareness of the malaria vaccine was determined using a linear regression model. The level of significance (alpha) was set to 0.05. In all analyses, the coefficients and standard errors were exponentiated for the ease of interpretation.

## 3. Results

The final sample included 3896 participants residing in three sub-Saharan African countries, including Uganda (*n* = 843), Ghana (*n* = 765), and Nigeria (*n* = 2288). Most of the respondents (58.3%) indicated that they were aware of the malaria vaccine. The distribution of the respondents’ awareness relative to the respective demographics, including age, gender, education, residential location, and country of residence was assessed (see Table 1).

By age category, the largest proportion of respondents were individuals between the ages of 25 and 34 years, constituting approximately 40.5% of the total respondents. This category also constituted the highest proportion of respondents in any age category indicating that they had heard of the malaria vaccine (59.5%). Most of the respondents across all age groups had heard of the malaria vaccine.

Overall, more male respondents (63.0%) had heard of the malaria vaccine compared to female respondents who were aware of the vaccine (53.1%). There were more individuals with an undergraduate degree who completed the survey, constituting approximately 55.5% of the respondents. Most of the respondents with a secondary school degree and lower had not heard of the malaria vaccine, while most of those with an undergraduate degree and higher had heard of the malaria vaccine. Most respondents (65.9%) were resident in large cities. For each demographic characteristic and category, respondents were more likely to be aware of the vaccine than not.

A linear regression model was used to determine the relationship between awareness of the malaria vaccine and each of the demographic characteristics in the different countries and overall, respectively (Table 2).

Gender and place of residence were significantly associated with public awareness of the malaria vaccine in Nigeria (*p* < 0.0001) and Uganda (*p* < 0.0001); with male respondents and urban residents being more likely to be aware, respectively. Age was a significant predictor of the public awareness of the malaria vaccine in Ghana (*p* = 0.0304); with younger respondents being more likely to be aware of the malaria vaccine. Overall, there was a significant association between public awareness and education status in all three sub-Saharan African countries assessed (*p* < 0.0001).

## 4. Discussion

The awareness of residents about the availability of lifesaving and disease preventative measures and public perception of these measures are critical to health outcomes expected from advancements in controlling the malaria epidemic in sub-Saharan Africa. The current study examined demographic factors, including age, gender, residence, and education status, relative to the awareness of the malaria vaccine.

From the survey data, the education status of respondents from the sub-Saharan African countries included (Nigeria, Ghana, and Uganda), was a significant predictor of the public awareness of the malaria vaccine. The current study findings are similar to some previous studies that found an association between vaccine awareness and education level. In the study by Voo and colleagues (2021), they found that among 405 parents who responded to their survey, awareness of childhood vaccines was increasingly higher with a higher education status [25]. Other studies also cite parental education level as being associated with an awareness and/or knowledge of childhood vaccines [26,27,28]. The positive correlation between awareness and education status could also be attributed to the fact that since most of the respondents were educated, they could be involved in information seeking and sharing among colleagues, increasing awareness in that demographic category [27]. The findings also highlight a critical gap, as lack of knowledge and awareness of childhood vaccines has also been associated with lower rates of childhood immunization [26,27,28]. Therefore, there must be an intentional focus on reaching less educated parents and caregivers with information regarding the malaria vaccine.

Literacy level in a society directly or indirectly plays a role in understanding health-related issues, whether interventional or preventive, and consequently influences health outcomes [29]. Low literacy is more prevalent in the low and middle-income countries which includes countries in sub-Saharan Africa [30]. In the current study, educational level was a predictor of malaria vaccine awareness among respondents in the sub-Saharan African countries surveyed. A lack of awareness of the malaria vaccine is a potential limitation to the uptake of the vaccine. This is in keeping with a study performed in Ghana by Tabiri et al. (2021), which showed that a higher level of education was a positive predictor of vaccine uptake [31]. This was because highly educated parents had access to information from multiple sources and were better placed to understand the details and essence of vaccination. A similar outcome was seen in Nigeria, where a mother’s educational level significantly contributed to her knowledge of three or more preventive measures against malaria [32]. The study also demonstrated that the level of awareness of the malaria vaccine was low among the respondents, and the level of education was a major contributory factor. Further evidence from Ghana concluded that caregivers who had less than secondary school education were less likely to complete their children’s immunization schedule compared with those who had a secondary education or higher [33,34]. The educational status of the mother was significantly associated with immunization status of the child, with the highest trend seen among mothers with tertiary education, respectively.

In this age of electronic information access, it is not surprising that younger persons, being more technologically oriented, were more aware of the malaria vaccine. Persons in the younger adult age categories are also more likely to be parents/caregivers of infants and children (ages 0–5 years) who are most affected by malaria mortality. It therefore follows that they would be more interested in keeping up with information regarding infant and child health. Health promotion efforts and messaging regarding the malaria vaccine should target this demographic. However, given the communal context of cultures in most sub-Saharan countries, older adults such as grandmothers must be reached with relevant information, as they are often integral to childcare and very influential with regard to health-related decisions in the home [35].

The initial vaccine rollout in Kintampo, formerly in the Brong Ahafo Region (now in Bono East Region) and Agogo in the Ashanti Region of Ghana, did not receive extensive media coverage. According to Asante K. P. et al., the pilot implementation was met with conspiracy theory rumors that were probably spread by anti-vaxxers [36]. Therefore, to ensure the progress and effective administration of the vaccine, vast media coverage seems to have been localized within the selected regions [37].

This study found a significant association between the awareness of the malaria vaccine and a place of residence for two (Nigeria and Uganda) of the three countries. The localization of information on the vaccine rollout and the prevalence of misinformation can account for the non-correlation in Ghana. The fact that there is approximately 50% access to a smartphone in Ghana, higher than what is obtainable in Nigeria (16.3%) and Uganda (18%), can explain the prevalence of digital misinformation in Ghana [38,39,40]. Additionally, Internet penetration in Ghana (53%) is more than that in Nigeria (51%) and Uganda (29.1%) [41,42,43]. Inequities in access to information on vaccines between residents in rural and urban settings have been shown to adversely affect population-wide childhood immunization coverage [44]. Generally, in the literature, a lack of targeted messaging, communication, and information have been cited as reasons for the non/under-vaccination of children in low-income and middle-income countries [45]. These findings present necessary incentives for country-specific policy reforms if universal immunization coverage with regard to malaria is to be achieved in sub-Saharan Africa.

## 5. Limitations

The current study utilized a convenience sampling technique. As the sampling was not random, the sample population is not representative of the study population. The fact that approximately only 19.8% (54.7 million) of the population have access to a smartphone [38,39,40] and approximately 0.01% of those with a smartphone completed the survey further limits the representativeness. The web-based survey had to be accessed on a device with Internet connectivity, which may have limited responses to those with devices having such capability (e.g., a smartphone, tablet, laptop, etc.) as well as Internet access. Given the significant disparities in mobile phone penetration and Internet access across rural–urban divides and socio-economic groupings [46], the online survey strategy adopted in this study was therefore limited by response bias. Mostly utilizing social media for dissemination of the survey link, is another source of limitation to the study, as there is a limit to which people can be reached with detailed information that encourages participation. The survey was self-administered in English only, hence respondents had to be literate and therefore more likely to be educated, further increasing the possibility of a significant difference by educational level. The opening statement of the survey served as information about the availability of the vaccine. This may have influenced the participants’ responses about their awareness of the malaria vaccine. Lastly, we did not assess how the respondents knew about the vaccine.

## 6. Conclusions

Beyond the availability of the malaria vaccine for use in malaria-endemic regions of the world, including sub-Saharan African countries, residents’ awareness and willingness to accept the vaccine are essential to reducing the malaria burden. Public awareness of the malaria vaccine is a critical part of all measures directed towards the treatment and management of the disease without replacing prevention strategies. Therefore, stakeholders of health, including individuals, providers, and healthcare professionals, public health professionals, health institutions and organizations, community-based organizations, faith-based organizations, and government at the local, state, and federal levels, must pay attention to evidence-based drivers of the public awareness of the malaria vaccine.

Given the negative impact of the lack of awareness and knowledge, misinformation and conspiracy theories on immunization programs, public health campaigns preceding the population-wide roll-out of the novel malaria vaccine should target the less-educated, and those residing in more rural areas. Culturally responsive efforts should target women of child-bearing age as well as the older women who are integral in childcare, while assuring equitable access to the malaria vaccine across sub-Saharan Africa.

## Figures and Tables

**Table 1:** Awareness of the malaria vaccine by demographic characteristics and categories.

Demographics Characteristics and Categories		Unaware of the Malaria Vaccine, N (% per Category)	Aware of the Malaria Vaccine N (% per Category)	Total no. of Respondents per Category (% of Respondents)
Age (years)	18–24	530 (43.1)	701 (56.9)	1231 (31.6)
	25–34	640 (40.5)	939 (59.5)	1579 (40.53)
	35–44	240 (41.0)	346 (59.0)	586 (15.04)
	45–54	152 (43.2)	200 (56.8)	352 (9.03)
	55–64	53 (43.8)	68 (56.2)	121 (3.11)
	65–74	10 (43.5)	13 (56.5)	23 (0.59)
	75–84	1 (33.3)	2 (66.7)	3 (0.078)
	85 or older	0 (0)	1 (100)	1 (0.027)
Gender	Female	875 (46.9)	992 (53.1)	1867 (47.9)
	Male	742 (37.0)	1265 (63.0)	2007 (51.5)
	Prefer not to say	9 (60.0)	6 (40.0)	15 (0.4)
	Other	0 (0)	7 (100)	7 (0.18)
Education status	No education	4 (50.0)	4 (50.0)	8 (0.2)
	Primary school	8 (57.1)	6 (42.9)	14 (0.4)
	Secondary school	178 (53.1)	157 (46.9)	335 (8.6)
	Undergraduate degree	926 (42.8)	1237 (57.2)	2163 (55.5)
	Graduate/professional degree	477 (36.9)	817 (63.1)	1294 (33.2)
	Other	33 (40.2)	49 (59.8)	82 (2.1)
Residence (population)	Hamlet (<100)	6 (31.6)	13 (68.4)	19 (0.5)
	Village (100–2499)	93 (47.9)	101 (52.1)	194 (5.0)
	Small town (2500–9999)	201 (46.2)	234 (53.8)	435 (11.2)
	Mid-sized town (10,000–49,999)	270 (39.7)	410 (60.3)	680 (17.4)
	Large city/metropolitan area (>50,000)	1056 (41.1)	1512 (58.9)	2568 (65.9)
Country of residence	Nigeria	1013 (44.3)	1275 (55.7)	2288 (58.7)
	Uganda	273 (32.4)	570 (67.6)	843 (21.6)
	Ghana	340 (44.4)	425 (55.6)	765 (19.7)

**Table 2:** Association between public awareness of the malaria vaccine and demographic characteristics.

Variables	*p*-Value			
	Nigeria	Ghana	Uganda	Sub-Saharan Africa
Awareness vs. age	0.1005	0.0304	0.0799	0.9331
Awareness vs. gender	<0.0001	0.0860	<0.0001	<0.0001
Awareness vs. residence	0.0185	0.8736	0.0002	0.0610
Awareness vs. education status	0.0336	<0.0001	0.0406	<.0001

## Data Availability

The data presented in this study are available on request from the corresponding author. The data are not publicly available due to privacy reasons.

## Abbreviations

WHO	World Health Organization
RDT	rapid diagnostic tests

## References

[1] World Health Organization Fact Sheet about Malaria. https://www.who.int/news-room/fact-sheets/detail/malaria.

[2] Aremu T.O., Ajibola O.A., Oluwole O.E., Adeyinka K.O., Dada S.O., Okoro O.N. (2022). Looking Beyond the Malaria Vaccine Approval to Acceptance and Adoption in Sub-Saharan Africa. Front. Trop. Dis..

[3] World Health Organization—WHO WHO Recommends Groundbreaking Malaria Vaccine for Children at Risk. https://www.who.int/news/item/06-10-2021-who-recommends-groundbreaking-malaria-vaccine-for-children-at-risk.

[4] WHO | Regional Office for Africa Malaria Vaccine Pilot Launched in Ghana. https://www.afro.who.int/news/malaria-vaccine-pilot-launched-ghana.

[5] Talisuna A., Adibaku S., Dorsey G., Kamya M.R., Rosenthal P.J. (2012). Malaria in Uganda: Challenges to control on the long road to elimination. II. The path forward. Acta Trop..

[6] World Malaria Report 2021, Regional Data and Trends. https://cdn.who.int/media/docs/default-source/malaria/world-malaria-reports/world-malaria-report-2021-regional-briefing-kit-eng.pdf?sfvrsn=338167b6_25&download=true.

[7] National Malaria Control Program Ministry of Health | Government of Uganda. https://www.health.go.ug/programs/national-malaria-control-program/.

[8] Balikagala B., Fukuda N., Ikeda M., Katuro O.T., Tachibana S.-I., Yamauchi M., Opio W., Emoto S., Anywar D.A., Kimura E. (2021). Evidence of Artemisinin-Resistant Malaria in Africa. N. Engl. J. Med..

[9] Ghana—PMI. https://www.pmi.gov/where-we-work/ghana/.

[10] Fighting Malaria Fights Poverty: Malaria Prevention in Ghana The Borgen Project. https://borgenproject.org/fighting-malaria-fights-poverty-malaria-prevention-in-ghana/.

[11] Nabarro D. (1999). Roll Back Malaria. Parassitologia.

[12] Ghana Buisness News—GBN Ghana Records 89% Drop in Malaria-Related Deaths in Eight Years. https://www.ghanabusinessnews.com/2021/04/29/ghana-records-89-drop-in-malaria-related-deaths-in-eight-years/.

[13] Onwujekwe O., Uguru N., Etiaba E., Chikezie I., Uzochukwu B., Adjagba A. (2013). The Economic Burden of Malaria on Households and the Health System in Enugu State Southeast Nigeria. PLoS ONE.

[14] Ahuru R.R., Iseghohi J.O. (2018). The economic burden of malaria, evidence from Nigeria’s data. Amity J. Healthc. Manag..

[15] Chijioke-Nwauche I., Maduka O., Awopeju A., Oboro I., Paul N., Ogoro M., Otto G., Kasso T., Yaguo-Ide L., Abam C. (2020). Malaria and Its Economic Burden among Pregnant Women in Rivers State, Nigeria. Open J. Obstet. Gynecol..

[16] Arrow K.J., Panosian C., Gelband H. (2004). The Cost and Cost-Effectiveness of Antimalarial Drugs.

[17] Coleman P.G., Shillcutt S., Morel C., Mills A.J., Goodman C. (2004). A Threshold analysis of the cost-effectiveness of artemisinin-based combination therapies in sub-saharan africa. Am. J. Trop. Med. Hyg..

[18] Talisuna A.O., Meya D.N. (2007). Diagnosis and treatment of malaria. BMJ.

[19] WHO (2010). Guidelines for the Treatment of Malaria.

[20] WHO (2018). World Malaria Report.

[21] Sonibare O.O., Bello I.S., Olowookere S.A., Shabi O., Makinde N.O. (2020). Effect of malaria preventive education on the use of long-lasting insecticidal nets among pregnant females in a Teaching Hospital in Osun state, south-west Nigeria. Parasite Epidemiol. Control.

[22] Okafor I.P., Ezekude C., Oluwole E.O., Onigbogi O.O. (2019). Malaria in pregnancy: A community-based study on the knowledge, perception, and prevention among Nigerian women. J. Fam. Med. Prim. Care.

[23] Chukwuocha U.M., Okorie P., Iwuoha G.N., Ibe S.N., Dozie I.N., Nwoke B.E. (2018). Awareness, perceptions and intent to comply with the prospective malaria vaccine in parts of South Eastern Nigeria. Malar. J..

[24] 6 African Nations Chosen for mRNA Vaccine Production | Devex. https://www.devex.com/news/6-african-nations-chosen-for-mrna-vaccine-production-102706.

[25] Voo J.Y.H., Lean Q.Y., Ming L.C., Hanafiah N.H., Al-Worafi Y.M., Ibrahim B. (2021). Vaccine Knowledge, Awareness and Hesitancy: A Cross Sectional Survey among Parents Residing at Sandakan District, Sabah. Vaccines.

[26] Papazoglou A., Giamaiou K., Poulopoulou S., Pavlopoulou I., Tsoumakas K. (2013). The National Vaccination Programme in Greece: Factors Affecting Parents’ Knowledge. Glob. J. Med. Res..

[27] Bernsen R.M., Al-Zahmi F.R., Al-Ali N.A., Hamoudi R.O., Ali N.A., Schneider J., Al-Mutawa J., Grivna M. (2011). Knowledge, Attitude and Practice towards Immunizations among Mothers in a Traditional City in the United Arab Emirates. J. Med Sci..

[28] Al-Zaharani J. (2013). Knowledge, Attitude and Practice of Parents Towards Childhood Vaccination. Majmaah J. Health Sci..

[29] DeWalt D.A., Berkman N.D., Sheridan S.L., Lohr K.N., Pignone M. (2004). Literacy and health outcomes. J. Gen. Intern. Med..

[30] Azevedo J.P. (2020). Learning Poverty: Measures and Simulations.

[31] Tabiri D., Ouédraogo J.C.R.P., Nortey P.A. (2021). Factors associated with malaria vaccine uptake in Sunyani Municipality, Ghana. Malar. J..

[32] Musa-Booth T., Enobun B., Agbomola A., Shiff C. (2021). Knowledge, attitude and willingness to accept the RTS,S malaria vaccine among mothers in Abuja, Nigeria. Ann. Afr. Med. Res..

[33] George A.A. (2017). Determinants of Complete Vaccination Among Children 24–35 Months in Ga East Municipality of Accra. University of Ghana Http://Ugspace.Ug.Edu.Gh Declaration. Ann Trop Paediatr..

[34] Ofosu S.K. (2017). Factors Contributing to Immunisation Coverage in Assin North Municilpality. Master’s Thesis.

[35] Michel J., Stuckelberger A., Tediosi F., Evans D., Van Eeuwijk P. (2020). The roles of a Grandmother in African societies—Please do not send them to old people’s homes. J. Glob. Health.

[36] Asante K.P., Binka F.N., Koram K.A. (2019). Malaria vaccine deployment in Africa: Focus on Ghana. Ghana Med. J..

[37] Ogutu B.R., Baiden R., Diallo D., Smith P.G., Binka F.N. (2010). Sustainable development of a GCP-compliant clinical trials platform in Africa: The Malaria Clinical Trials Alliance perspective. Malar. J..

[38] Smartphone Users in Nigeria 2014–2025 | Statista. https://www.statista.com/statistics/467187/forecast-of-smartphone-users-in-nigeria/.

[39] Statista Uganda Number of Smartphones 2020. https://www.statista.com/statistics/1082286/number-of-smartphones-registered-in-uganda/.

[40] Digital in Ghana: All the Statistics You Need in 2021 DataReportal—Global Digital Insights. https://datareportal.com/reports/digital-2021-ghana.

[41] Statista Nigeria Internet User Penetration 2027. https://www.statista.com/statistics/484918/internet-user-reach-nigeria/.

[42] Digital 2022: Uganda DataReportal—Global Digital Insights. https://datareportal.com/reports/digital-2022-uganda.

[43] Digital 2022: Ghana DataReportal—Global Digital Insights. https://datareportal.com/reports/digital-2022-ghana.

[44] Naeem M., Khan M.Z.U.I., Adil M., Abbas S.H., Khan A., Naz S.M. (2011). Inequity in childhood immunization between urban and rural areas of Peshawar. J. Ayub Med. Coll. Abbottabad JAMC.

[45] Rainey J.J., Watkins M., Ryman T.K., Sandhu P., Bo A., Banerjee K. (2011). Reasons related to non-vaccination and under-vaccination of children in low and middle income countries: Findings from a systematic review of the published literature, 1999–2009. Vaccine.

[46] Houngbonon G.V., Le Quentrec E., Rubrichi S. (2021). Access to electricity and digital inclusion: Evidence from mobile call detail records. Humanit. Soc. Sci. Commun..

